# The green shoots of a novel training programme: progress and identified key actions to providing services to MSM at Kenyan health facilities

**DOI:** 10.7448/IAS.18.1.20226

**Published:** 2015-10-21

**Authors:** Elise M van der Elst, Bernadette Kombo, Evans Gichuru, Anisa Omar, Helgar Musyoki, Susan M Graham, Adrian D Smith, Eduard J Sanders, Don Operario

**Affiliations:** 1 Kenya Medical Research Institute – Wellcome Trust Research Programme, Kilifi, Kenya; 2 County Ministry of Health, Kilifi, Kenya; 3 National AIDS and STI Control Programme, Nairobi, Kenya; 4 Departments of Medicine, Epidemiology and Global Health, Washington University, Seattle, WA, USA; 5 Nuffield Department of Population Health, Oxford University, Oxford, UK; 6 School of Public Health, Brown University, Providence, RI, USA

**Keywords:** MSM sensitivity and skills training, healthcare provider, stigma, MSM, Kenya, HIV

## Abstract

**Introduction:**

Although men who have sex with men (MSM) in sub-Saharan Africa are at high risk for HIV acquisition, access to and quality of health and HIV services within this population are negatively affected by stigma and capacity within the health sector. A recently developed online MSM training programme (www.marps-africa.org) was shown to contribute to reductions in MSM prejudice among healthcare providers (HCPs) in coastal Kenya. In this study, we used qualitative methods to explore the provision of MSM healthcare services two years post-training in coastal Kenya.

**Methods:**

From February to July 2014, we held 10 focus group discussions (FGD) with 63 participants, including HCP from 25 facilities, county AIDS coordinators and MSM from local support groups. Participants discussed availability, acceptability and accessibility of HIV healthcare for MSM. HCP also discussed changes in their health service practices after completing the training. FGD were recorded, transcribed verbatim and analyzed using Ritchie and Spencer's “framework approach” for qualitative data.

**Results:**

HCPs described continued improvements in their ability to provide service in a non-stigmatizing way to MSM patients since completing the training programme and expressed comfort engaging MSM patients in care. Four additional recommendations for improving MSM healthcare services were identified: 1) expanding the reach of MSM sensitivity training across the medical education continuum; 2) establishing guidelines to manage sexually transmitted anal infections; 3) promoting legal and policy reforms to support integration of MSM-appropriate services into healthcare; and 4) including MSM information in national reporting tools for HIV services.

**Conclusions:**

Positive impacts of this sensitivity and skills training programme were reflected in HCP attitudes two years post-intervention. Scaling-up of efforts will rely on continued policies to include MSM in healthcare programmes to reduce stigma in health settings and guidelines for MSM STI service delivery.

## Introduction

Men who have sex with men (MSM) make up a large proportion of new HIV infections in sub-Saharan Africa (SSA) [1–6]. Yet MSM face challenges accessing HIV health services, and investment in targeted, knowledgeable and sensitive services is urgently needed [7–10]. Experiences of discrimination at health facilities – such as healthcare providers’ (HCPs’) stigmatizing attitudes and reluctance to talk about sexual matters – often result in substandard healthcare provision and can intensify MSM's fear of seeking healthcare [11–13]. In addition, polemic national debates about the morality of same-sex practices in Africa cast negative attention on MSM, raising barriers even higher for HCPs to provide quality healthcare to MSM and contributing to further difficulties in healthcare access among MSM [9,14–16]. Reports from the Global Commission on HIV and the Law seek to challenge stigma and promote inclusion in health policy for homosexuals, urging African governments and regional institutions to ensure access to HIV prevention, care and support for all people [17,18]. Implementation of recommendations from the commission remains a daunting task.

In Kenya, all citizens have the right to healthcare services in accordance with non-discrimination laws stated in the revised 2010 Kenyan national constitution [19], and the Kenyan Ministry of Health recently recognized MSM as a key population in their National AIDS Strategic Plan [20]. In 2013, the National AIDS and STI Coordination Programme (NASCOP) and the National AIDS Control Council committed to scaling-up equitable HIV services for MSM [21]. However, despite improvements in health policy, many Kenyan HCPs lack the training and skills to implement appropriate and non-discriminatory services to MSM patients in their facilities [9,22].

A recent pilot study conducted by NASCOP and researchers from the Kenya Medical Research Institute (KEMRI) sought to implement and evaluate an online education programme on treatment of MSM patients, developed specifically for HCPs with a clinical role, such as clinicians and nurse counsellors, in SSA (www.marps-africa.org). The programme was called the *MSM-Appropriate Services and Training*, or MAST programme. It consisted of eight self-administered modules delivered via computer and involved group discussions to facilitate peer support among HCPs for providing appropriate and non-judgmental HIV and STI services to MSM patients [23]. Since the launch of the open-access, web-based programme in December 2010, 1150 Kenyan HCPs have completed the MSM online training programme [24].

In 2012, an initial evaluation of the programme was conducted among 71 HCPs, of whom 52 had a clinical role. Findings showed that at three months post-training, more HCPs had acceptable levels of knowledge of MSM sexual health issues and lower levels of homophobic attitudes compared to baseline; effects were strongest among HCPs with high homophobia scores at baseline [8]. However, questions remain regarding the longer-term effect of this brief training intervention upon attitudes and professional practices towards MSM patients among trained HCPs [25].

This paper explores attitudes and professional practices of a subset of the HCPs in clinical roles two years after completing the training. Qualitative methodology was used to explore HCP experiences with MSM patients, perceptions about gaps in service provision for MSM and beliefs about strategies to maximize the quality of care for MSM patients. Narrative experiences from members of the health policy sector and MSM community members also provided insight into health services for this population.

## Methods

### Participants and procedures

The study was conducted between February and July 2014 in Kilifi and Mombasa counties in coastal Kenya. Qualitative methods were used to collect narrative data about participants’ perceptions and experiences about the provision and quality of local health services for MSM. Three groups of respondents were recruited: 1) HCPs who completed the original MSM online training programme two years ago, presently providing clinical services to MSM in the study area; 2) MSM involved in local community-based MSM organizations (CBOs) and 3) local policymakers, referred to as *county AIDS and STI coordinators* (CASCOs), willing to discuss policy and health service practices for MSM. Due to small numbers of CASCOs, we conducted paired interviews each with two CASCOs from different districts; all others participated in focus group discussions (FGDs).

The study procedures were approved by the ethical review board at the KEMRI and all participants provided written informed consent. HCPs and CASCOs received 1000 Kenyan shillings (approximately US$12) for travel and time compensation, and MSM were offered lunch and 500 Kenyan shillings. Reimbursement amounts were determined based on previous studies with these groups and were deemed appropriate and non-coercive according to local standards.

### Focus groups and interviews

Out of the 52 HCPs who had previously participated in the pilot study, 32 HCPs still provided clinical services in the study area and participated in five FGDs (six to eight participants per group). Twenty HCPs were considered lost to follow-up partly due to administrative transformation with the promulgation of Kenya's new constitutions. HCPs either had migrated back to their home counties or were transferred outside the study area. Three FGDs were held with 31 local MSM community group members, known by their organizations to frequent sensitized healthcare facilities in the coast (9 to 11 participants per group). Four CASCOs from the administrative units along the coast were invited for paired interviews. The average age of the HCPs and CASCOs was 38 years, 50% were male, 20% Muslim and 80% Christian. The average age of the MSM participants was 26 years, 50% were Muslim, 84% unemployed and 55% had completed secondary education.

Following on from discussion of data from the pilot study's focus groups, which elicited HCPs’ overall reaction to the sensitivity and skills training programme [9], discussion topics focused on participants’ experiences during the two years following delivery of the MSM sensitivity training programme. Themes included perceptions and experiences about local healthcare delivery for MSM and perceptions and experiences of healthcare services for MSM in terms of availability, quality and accessibility. Discussions were semi-structured and facilitated by a member of the research team, with a co-facilitator present to observe and take notes.

Discussions with HCPs and CASCOs were conducted in English; discussions with MSM participants were mainly in Kiswahili depending on preference and language skills. All discussions were audiotaped, transcribed verbatim and entered into NVivo. FDGs conducted in Kiswahili were translated into English by two members of the research team.

Analyses of qualitative data followed the “framework approach” described by Ritchie and Spencer [26], which involves systematic coding to identify and define concepts emerging from the data, mapping the concepts, creating typologies, finding associations between concepts and seeking explanations from the data. Data were coded and triangulated by two colleagues independent of the interviews to ensure that interpretations of quotes were consistent and that data analysis was rigorous and transparent.

## Results

Expanding the reach of MSM sensitivity training:

### Professional transformations

Two years post-training, HCPs described how programme participation influenced their ongoing ability to provide non-judgmental health services to MSM patients. They described refraining from making professional judgments of MSM patients based on personal opinions and values, and they remarked on transformations in their ability to integrate new knowledge and skills about HIV and other STIs into their professional practice with MSM.

Before, I had a negative attitude towards MSM, but after the training I realized that they are human beings. I should accept them the way they are. I changed my attitude, and when the guys saw me they said, “So you went for that [sensitivity] training, are you ready to work with us?” I said “Yes, don't worry ….” (nurse, 38, Muslim, female)

After the training I realized it's my work to reduce harm. Now I can treat without discrimination … I told him [MSM patient] “If you are not comfortable, please come and see someone that you'll be free with, and be helped with ART adherence” … and he did come back and was started on medication. (nurse, 32, Christian, male)

As the latter quote suggests, increased capacity to provide non-stigmatizing and non-discriminatory services to MSM patients can contribute to critical HIV outcomes, including engagement in care and ART adherence.

HCPs noted that a key component of the MAST programme was the opportunity to share with colleagues their experiences in providing services to MSM patients and reflect on their challenges in providing effective care, as noted in this quote:

For the first time we were actually talking about giving services to homosexuals. So in a way, we stopped looking at them as people who are not in our thinking … and tried to reflect on the many times we may have misdiagnosed patients with anal pain assuming it's hemorrhoids. (clinician, 37, Christian, female)

Thus, not only did programme participants benefit from new knowledge and skills provided to HCP trainees, but they gained a safe context for discussion and reflection about the quality of their services to MSM.

### Integration of MSM sensitivity training in medical education

HCPs recommended the inclusion of MSM sensitivity training as an integrated component of medical education in Kenya. They recommended that such training be integrated along the medical educational continuum, from undergraduate curriculum to postgraduate training and lifelong learning. There was recognition that MSM sensitivity training is particularly needed at the pre-service level:

We should lobby and ensure that MSM sensitivity training is incorporated in the curriculum at pre-service level. None of these things are in the syllabus of any medical school: these are things that come and you find them as you work. (clinician, 35, Christian, male)

Participants remarked that integrating MSM sensitivity training into medical education would allow trained HCPs to more easily recognize the presence of MSM patients in their care and empathize with MSM patients who experience difficulty presenting their health concerns. For example, a participant commented on a tendency for MSM to speak about their same-sex behaviour using innuendo rather than direct language, which might confuse non-trained HCPs:

Someone [HCP colleague] was sharing with me that a patient came with an STI, but it took a while before that person could explain. The patient kept on saying: “You know I am not like you”, and the HCP was like: “If you are not like me then who are you?”, and he insisted “I am not a man like you”, but the HCP still couldn't get it until we talked about it. Thank God it was after the training. (clinician, 39, Christian, female)

### Muslim HCPs and younger male HCPs

Providing training to HCP with Muslim backgrounds was highlighted as a particularly challenging part of scaling-up the training and integrating MSM sensitivity into local practice. For example, a young Muslim HCP expressed difficulty implementing new skills following participation in the MAST programme:

I was told, “Now you as a Muslim what is this you are bringing to us? Where will you begin here, yet you know we are in a Muslim neighborhood?” It wasn't easy, but since I had the MSM sensitivity guide I shared with them, okay, some Muslim HCPs picked it, but not all … some of them will never change no matter how you try …. (clinician, 32, Muslim, male)

Similarly, training young male HCPs is a challenge due to homophobia and fears of secondary stigma among young heterosexual men. This issue is reflected in two quotes from the perspective of an MSM community member and an HCP:

Then, there is something that we have come to realize, when we weigh the HCPs who are friendly to MSM. Young males are the most homophobic. I think it is because they [young male HCPs] have big egos, and fear of being labelled ‘husbands of gays’. (MSM, 25, Muslim, unemployed, sec school)

When he [MSM patient] came to me, I didn't want him to be stigmatized, I walked out with him at the corridors and we sat and talked. But the way my co-workers looked at me until that procedure was over … at first I couldn't function. I talked to that guy and made sure he got his medication, but after he had left I was stigmatized, people didn't even want to come close to me. (clinician, 29, Muslim, male)

### Sustaining change and scaling-up the sensitivity training programme

Sustaining the outcomes from the MSM sensitivity training programme was a noted concern, because many of the original trainees had left the region in the two years since the programme ended. CASCOs remarked on understaffing, heavy workloads and lack of government support as factors that contribute to high staff turnover that, ultimately, can minimize long-term results:

The majority of the staff we trained went elsewhere. Some resigned, some joined other institutions and some went back to their mother county following the devolution (The term *devolution* refers to the statutory granting of powers from Kenya's central government to a new subnational level of government. This administrative change took place in 2012 and caused the outmigration of HCPs, who returned to the counties they originated from). Then some were internally transferred. In line with this, who will be responsible to continuously keep the sensitivity training going? The national government, the county, HCPs themselves? (CASCO, 39, Muslim, male)

Efforts to expand the scope of MSM sensitivity training outside of the health sector were noted as a strategy to improve societal inclusion of MSM individuals. For example, the following quote suggests a need to provide MSM sensitivity training to law enforcement, policymakers and religious leaders, in order to truly broaden the reach of the programme:

In the training, we focused on the HCPs, but we need to identify other service providers MSM need their services from, like the police, local administration, or religious leaders and train them so that they can support MSM access of services and deal with stigma in the community …, we need to bring a holistic approach in this access of services for MSM. (CASCO, 45, Muslim, female)

### Need for national guidelines to manage sexually transmitted anal infections

HCPs described how the absence of guidelines on management and treatment of anal STIs limited their ability to correctly diagnose anal STIs.

You know, we have this syndromic management of STIs, but it focuses on the vagina and urethra, there is none that has treatment for anal conditions. That's why the training was an eye opener, especially the way we ignore anal STIs. When a man comes in and says “*naumwa huko chini*” [I have pain down there] no one takes time to find out “*chini wapi*?” [down where?]. We all assume it is hemorrhoids and take him for surgery. (clinician, 38, Christian, female)

Because of time pressure and staff shortages, some clinicians admitted to superficial discussion with MSM patients. In government facilities, where patient loads are particularly high, HCPs described lacking the energy or enthusiasm to ask about sexuality or STI symptoms, failing to take sexual history and declining sexual examinations. HCPs felt that clear guidelines on diagnosing and managing anal STIs would give impetus for increased attention to relevant symptoms. One clinician remarked:

… you know, some of our colleagues are very rough and they don't follow the right procedures. They'll be like “say your problem, and if you don't want to … just go ….” They want to clear the queue and don't have time for ‘particular sensitive clients’. If we could have procedures on how to go about sexual history, and physical examination, we'll be able to identify anal STIs, and give effective medication. (clinician, 35, Christian, male)

### Need for legal and policy reforms

CASCOs called for a paradigmatic change, whereby the code of ethics stated in national policies needs to be embedded in a broader spectrum of HCP professional and personal competencies. They emphasized the constitutional mandate of HCPs with regard to offering healthcare services as a basic human right:

In our health sector strategic plan, we also have the code of regulations and codes of ethics. They are written all over in the health facilities. As the county health management team we are taking our staff through the new constitution with regard to health, but I think we need to have more forums to deliberate and have open discussions [to support provision of services to MSM]. (CASCO, 43, Muslim, male)

CASCOs remarked on the tension and legal uncertainty among HCPs with respect to providing healthcare to MSM – i.e. the fear of contravening the constitution, which criminalizes same-sex relationships, contrasted with the duty to obey legal mandates to provide non-discriminatory healthcare services.

The constitution talks about same-sex as illegal, and then when you go to the same constitution it talks about provision of services without discrimination … “service to all” and “*huduma bora ni haki yako*” [better services are your right]. What are we supposed to do? (CASCO, 38, Christian, female)

CASCOs called for both advocacy and paralegal educational programmes for health staff. Moreover, they felt that medical educators and chief healthcare officers and administrators have a distinct responsibility in implementing national policy:

You look at anything that happens in an institution … You see when you are in charge of a team it is something that transcends right from the big person, the person with authority who is a role model. (CASCO, 39, Muslim, male)

### Inclusion of MSM in implementation and reporting tools

The lack of inclusion of MSM in service implementation and reporting tools proved to be an inhibiting factor to monitoring and evaluating MSM services. CASCOs recommended improving national reporting instruments by recognizing and including MSM:

… we can come up with a sample [monitoring and evaluation tool] that we use to report with NASCOP, then … eventually when they [superiors] see the need they can fine tune it for national usage. (CASCO, 38, Christian, female)

CASCOs also suggested conducting regular audits of the availability of comprehensive HIV packages for MSM, including lubricants and provision of proctoscopies at government and private healthcare facilities. To improve provision of appropriate services, CASCOs discussed creating specific standard operating procedures (SOPs) to monitor quality control and quality assurance when treating MSM patients:

We probably need to come up with SOPs. It can outline exactly what is needed and help with planning and quantification of commodities and equipment to be used. (CASCO, 43, Muslim, male)

Similarly, MSM community members remarked on the need to develop rating systems for healthcare facilities based on the experiences of MSM patients. Health service rating tools could assess MSM patients’ general appraisals of HCP skills as well as patient access to specific services such as anal STI screening:

There are praises we give out there about clinics. Without that, peers (MSM) won't know about X (a MSM friendly health facility). (MSM, 26, Christian, unemployed, sec school)

According to the CASCOs, other changes must also be documented such as the emergence of MSM support groups led by trained HCPs:

We never used to have MSM support groups, but now we do. And the people manning these support groups are the nurses and some of the clinical officers who underwent the sensitivity training. They seem to be comfortable interacting with MSM and talking about HIV-MSM related issues. (CASCO, 45, Muslim, female)

Collecting information about MSM services in implementation and reporting tools thus allow for enhanced documentation of patient outcomes, which is essential for monitoring the ongoing effectiveness of HIV programmes for key risk populations.

## Discussion

Lessons learned related to the general positive effect of training on HCP attitudes and competencies towards serving MSM patients and the need for additional work on operational policies at the national and decentralized levels. The two-year post-assessment was able to monitor progress, such as HCPs’ competence to openly talk about HIV- and MSM-specific issues, coupled with non-judgmental attitudes, and to also outline key actions necessary to ensure supportive policies, such as governmental catalyzers recommending MSM pre-service and in-service curricula, and MSM non-discriminative policies throughout the public health system. Sustained impact of the MAST programme will rely on deliberate efforts and a continued collaborative approach to providing healthcare for MSM, based on the premise that teamwork among HCPs and learning from others were central to creating the conditions for culturally competent services. Institutional capacity building and sustainability were marked as essential to foster policy and programme development on a lasting basis, as were regular assessments (such as these) to continuously inform plans related to improvement.

A remaining challenge for HCPs is the confusion about developing and maintaining professional attitudes towards all patients, despite criminal laws making same-sex behaviour illegal in Kenya. Consistent with the findings on MSM health services in Malawi by Wirtz *et al*. [27], HCPs and CASCOs in coastal Kenya described a tension between the criminalization of same-sex practices versus the professional imperative to provide patient care and risk-reduction counselling. While it is unlikely that punitive laws will change in the near future, clearer guidance on this legal conflict should be provided by national programming initiatives. If non-judgmental healthcare services for MSM are to become the standard of care, continuous reinforcement from higher authorities is needed to support appropriate service implementation by HCPs. Moreover, to promote tolerance and inclusion of MSM in the broader society, MSM sensitivity training must be provided to other sectors including law enforcement, religious institutions and local government. In addition, the results of this study underscore the importance of combining processes such as extension of sexual health education for HCPs, expansion of clinical guidelines and greater inclusion of MSM behaviour characteristics in reporting tools as addressed in a recently developed model (SPEND) by Ross *et al*. [28].

A systematic review of the published literature on MSM in Africa since 2011 identified remarkably few studies on community engagement with MSM [29]. How often, with whom and what precisely constitutes MSM community engagement should be a topic of research to enhance healthcare for MSM [29]. A range of strategies to raise awareness and sensitize the wider community about MSM experiences is also needed. A recent study from coastal Kenya suggests that watching a brief film about the experiences of MSM and participation in a follow-up discussion contributed to attitude change towards MSM in a diverse community sample [30]. Efforts to increase experiential learning, guided support and positive intergroup contact can bolster the community context for improved health and wellness among MSM.

Limitations to this research must be acknowledged. First, participants in this sample might have been prone to socially desirable reporting about their attitudes and experiences related to health services for MSM. In addition, selection bias of HCPs might have influenced findings, as HCPs were included on basis of their experiences serving MSM patients and they may have had favourable attitudes towards MSM. The same applies for the sample of MSM participants as selected; MSM CBO members might not have represented the views of MSM who are not members of a CBO. Furthermore, the findings reported here do not permit temporal, causal or quantitative inferences. Finally, because this study took place in a region where MSM research has occurred for over nine years, findings might not be generalizable in other areas in Kenya or SSA.

## Conclusions

Incremental improvements in the ability to offer MSM healthcare services were reported two years post-intervention. Stigma towards MSM was still prevalent according to this diverse range of participants, and enhanced rollout of MSM sensitivity training was recommended for various cadres of healthcare staff, most notably superiors and managers, as well as young male HCPs. Involvement with the broader community was noted as a strategy to enhance general social inclusion of MSM. CASCOs advocated for reporting tools inclusive of MSM populations in terms of programme implementation and capacity building, and HCPs expressed the need for updated national guidelines to manage anal STIs. Across groups, participants felt that the national government should endorse MSM sensitivity training in order for HCPs to provide appropriate professional conduct in healthcare and HIV services.

## Competing interests

The authors declare that they have no competing interests.

## Authors' contributions

EMvdE, EG, AO, HM, SMG, ADS, EJS and DO contributed significantly to the study design. EMvdE and EJS conceived the study. BK conducted the FGDs and interviews. EMvdE and BK analyzed the data. EMvdE, DO, SMG and EJS discussed full texts. EMvdE drafted the manuscript. DO, SMG and ADS critically edited the manuscript. All authors read and approved the manuscript. DO is the guarantor of the paper.
